# Health-Related Quality of Life and Internalising Symptoms in Romanian Children with Congenital Cardiac Malformations: A Single-Centre Cross-Sectional Analysis

**DOI:** 10.3390/healthcare13151882

**Published:** 2025-08-01

**Authors:** Andrada Ioana Dumitru, Andreea Mihaela Kis, Mihail-Alexandru Badea, Adrian Lacatusu, Marioara Boia

**Affiliations:** 1 Doctoral School, “Victor Babes” University of Medicine and Pharmacy Timisoara, 300041 Timisoara, Romania; andrada.dumitru@umft.ro; 2 Research Center for Pharmaco-Toxicological Evaluations, Faculty of Pharmacy, “Victor Babes” University of Medicine and Pharmacy Timisoara, 300041 Timisoara, Romania; 3 Dermatology Department, The George Emil Palade University of Medicine, Pharmacy, Science, and Technology, 540139 Targu Mures, Romania; 4 Department of Pediatrics, “Victor Babes” University of Medicine and Pharmacy Timisoara, 300041 Timisoara, Romania; lacatusu.adrian@umft.ro; 5 Discipline of Neonatology, “Victor Babes” University of Medicine and Pharmacy Timisoara, 300041 Timisoara, Romania; boia.marioara@umft.ro

**Keywords:** heart defects, quality of life, child, depression, anxiety disorders

## Abstract

**Background and Objectives**: Although survival after congenital cardiac malformations (CCM) has improved, little is known about Romanian children’s own perceptions of health-related quality of life (HRQoL) or their emotional burden. We compared HRQoL, depressive symptoms, and anxiety across lesion severity strata and explored clinical predictors of impaired HRQoL. **Methods**: In this cross-sectional study (1 May 2023–30 April 2025), 72 children (mean age 7.9 ± 3.0 years, 52.8% male) attending a tertiary cardiology clinic completed the Romanian-validated Pediatric Quality of Life Inventory (PedsQL), Children’s Depression Inventory (CDI) and the Screen for Child Anxiety-Related Emotional Disorders questionnaire (SCARED-C, child version). Lesions were classified as mild (*n* = 22), moderate (*n* = 34), or severe (*n* = 16). Left-ventricular ejection fraction (LVEF) and unplanned cardiac hospitalisations over the preceding 12 months were extracted from electronic records. **Results**: Mean PedsQL total scores declined stepwise by severity (mild 80.9 ± 7.3; moderate 71.2 ± 8.4; severe 63.1 ± 5.4; *p* < 0.001). CDI and SCARED-C scores rose correspondingly (CDI: 9.5 ± 3.0, 13.6 ± 4.0, 18.0 ± 2.7; anxiety: 15.2 ± 3.3, 17.2 ± 3.8, 24.0 ± 3.4; both *p* < 0.001). PedsQL correlated positively with LVEF (r = 0.51, *p* < 0.001) and negatively with hospitalisations (r = −0.39, *p* = 0.001), depression (r = −0.44, *p* < 0.001), and anxiety (r = −0.47, *p* < 0.001). In multivariable analysis, anatomical severity remained the sole independent predictor of lower HRQoL (β = −8.4 points per severity tier, *p* < 0.001; model R^2^ = 0.45). Children with ≥ 1 hospitalisation (*n* = 42) reported poorer HRQoL (69.6 ± 8.0 vs. 76.1 ± 11.1; *p* = 0.005) and higher depressive scores (*p* < 0.001). **Conclusions**: HRQoL and internalising symptoms in Romanian children with CCM worsen with increasing anatomical complexity and recent hospital utilisation. The severity tier outweighed functional markers as the main determinant of HRQoL, suggesting that psychosocial screening and support should be scaled to lesion complexity. Integrating the routine use of the Romanian-validated PedsQL, CDI, and SCARED-C questionnaire into cardiology follow-up may help identify vulnerable patients early and guide targeted interventions.

## 1. Introduction

Congenital cardiac malformations (CCMs) remain the single largest category of major birth anomalies occurring in ≈ 8–12 per 1000 live births worldwide and in 9.6 per 1000 across Eastern European registries [1,2,3]. EUROCAT surveillance from 2008 to 2015 showed a stable total prevalence of 57.1 per 10,000 births, although rates varied markedly between national registries, reflecting true geographic heterogeneity and differences in prenatal detection policies.

Therapeutic advances have dramatically altered prognosis: the contemporary modelling of birth-cohort data suggests that >85% of children with even moderate–complex lesions now survive into adulthood, creating an expanding population of approximately 1.3 million European survivors who require lifelong follow-up [4]. Yet survival gains have not fully translated into normal functioning. Cross-sectional surveys of infants and toddlers awaiting repair already demonstrate clinically important deficits across all PedsQL™ domains [5], and a meta-analysis of surgical survivors reveals persistent impairments compared with healthy peers, with the largest effect sizes in physical and school functioning [6].

Large observational cohorts confirm that poorer health-related quality of life (HRQoL) is most pronounced in children with more complex anatomy and residual haemodynamic lesions, but psychosocial dimensions are also affected irrespective of physiology. A Dutch–German review identified anatomical severity, exercise intolerance, and recurrent symptoms as the strongest correlates of worse HRQoL in 5- to 18-year-olds [7], while a recent systematic review shows that children with severe CCM exhibit higher rates of withdrawal, depression, and attention problems than those with milder forms [8]. Moreover, pooled analyses across seven chronic paediatric conditions, including CCM, indicate that anxiety risk rises linearly as generic PedsQL scores decline, emphasising the entwined nature of physical limitation and emotional distress [9].

Meta-analytic data estimate that 30–40% of paediatric cardiac patients meet criteria for an internalising disorder—approximately triple the rate in healthy peers [10]. Neurocognitive profiling links depression and anxiety with poorer perceptual-organisational abilities and executive dysfunction in school-aged CCM survivors [11]. Family context adds another layer: parents of very young children with CCM report compromised family and child QoL soon after diagnosis [12], and a systematic review of 30 studies shows elevated parental anxiety, post-operative stress, and depressive symptoms during the first post-surgical year [13].

Health service use interacts bidirectionally with psychosocial status. Telemedicine follow-up has been shown to reduce parental anxiety and depression while improving perceived QoL [14]. In adults with CCM, brief cognitive-behavioural interventions yield small-to-moderate reductions in depressive symptoms [15]; similar principles underpin the CardioActive paediatric rehabilitation programme that combines supervised exercise with metacognitive anxiety management [16]. Conversely, frailty—often precipitated by repeated hospitalisations—affects one-third of post-Fontan adolescents and is independently associated with lower physical functioning scores and increased healthcare utilisation [16].

Robust assessment hinges on culturally validated instruments. Romanian versions of the PedsQL questionnaire demonstrate excellent feasibility and internal consistency (α > 0.70) in cohorts with chronic disease [17]. The Romanian SCARED-C likewise shows good internal consistency (α 0.63–0.91) and test–retest reliability (r = 0.70) [18]. For depressive symptoms, cross-cultural measurement invariance studies the support for the use of the Revised Child Anxiety and Depression Scale/Children’s Depression Inventory items in Southeastern European samples, including Romania [19].

Yet no Romanian investigation has simultaneously examined lesion complexity, ventricular performance, and recent hospital use as determinants of psychosocial health. We therefore hypothesised that anatomical severity would remain the principal, independent predictor of HRQoL after adjusting for cardiac function and hospitalisations. Accordingly, the present study sought to (i) compare HRQoL, depressive, and anxiety symptoms across mild, moderate, and severe CCM; (ii) examine associations between HRQoL and age, LVEF, and recent hospitalisation; and (iii) identify independent predictors of lower HRQoL in Romanian children, thereby informing severity-stratified psychosocial interventions.

## 2. Materials and Methods

### 2.1. Study Design and Ethics

This single-centre, observational, cross-sectional study was performed between May 2023 and April 2025 at the Timiş County Emergency Clinical Hospital “Pius Brinzeu”. Ethical approval was obtained from the institutional review board (IRB) of the hospital (Approval ID: 392 from 25 April 2023) in accordance with the Declaration of Helsinki. Written informed consent was obtained from all caregivers before enrolment, emphasising confidentiality and voluntary participation. Written informed consent was obtained from parents or legal guardians, and assent from children aged ≥ 7 years in accordance with the Declaration of Helsinki and GDPR. All data were anonymised and stored on encrypted, password-protected servers accessible only to study personnel.

### 2.2. Participants and Clinical Data Collection

Eligible participants were consecutive children aged 3–15 years with structurally confirmed CCM attending routine outpatient follow-up. Exclusion criteria were recognised syndromic diagnoses associated with neurodevelopmental delay, scheduled surgery within eight weeks, or insufficient Romanian language skills. Of the 76 eligible families approached, 4 declined participation, yielding 72 analysable cases (participation rate: 94.7%).

Demographic details (age, sex, and rural/urban residence) and clinical variables (malformation type, prior surgery, LVEF%, and the number of emergency cardiology-related admissions in the preceding 12 months) were abstracted from electronic records by two cardiologists blinded to the questionnaire results. Lesion complexity was categorised per the 2018 ACC/AHA guidelines [20] as mild (e.g., isolated small atrial septal defect), moderate (e.g., repaired tetralogy with residual lesions), or severe (e.g., single-ventricle Fontan circulation). This anatomical taxonomy is concordant with the 2020 ESC guidance [21] and facilitates cross-study comparison.

### 2.3. Instruments

PedsQL™ 4.0 Generic Core Scales (Romanian child self-report). This 23-item instrument covers physical, emotional, social, and school domains. Items are scored 0 = “never” to 4 = “almost always”; scores are reverse-transformed to 0–100, with higher values indicating better HRQoL. Physical and psychosocial summary scores and a total score (mean of all items) were computed. Romanian validation showed Cronbach’s α ≥ 0.88 across domains [17].

Children’s Depression Inventory (CDI-2)–long form, Romanian version. The CDI contains 27 symptom statements, each scored 0–2, producing a total range of 0–54 (higher = greater depressive severity). A cut-off ≥ 19 suggests clinically relevant depression. Romanian psychometrics demonstrate α 0.84–0.90 and good convergent validity with clinical interviews [22].

Screen for Child Anxiety-Related Emotional Disorders (SCARED-C) questionnaire, Romanian child version. The 41-item SCARED-C assesses generalised, separation, social, and school anxiety plus somatic/panic symptoms. Items are rated 0–2, and the total score is in the range of 0–82. A threshold ≥ 25 was retained because Romanian validation work demonstrated the highest Youden index at this score (sensitivity 0.81; specificity 0.77) [18].

### 2.4. Statistical Analysis

Analyses were conducted with SPSS v27. Normality was assessed using Shapiro–Wilk tests. Continuous variables approximated normal distribution; results are presented as the mean ± SD. One-way ANOVA compared severity groups, with Bonferroni post hoc adjustment when omnibus values were *p* < 0.05. Kruskal–Wallis tests confirmed robustness for skewed variables (hospitalisations). Pearson coefficients evaluated bivariate associations between the PedsQL total score and predictors.

A multivariable linear regression model was used, and variables were entered that demonstrated values *p* ≤ 0.10 during bivariate testing (severity tier coded 0–2, LVEF, hospitalisations, age). Multicollinearity was verified by a variance inflation factor < 2. Independent-samples t tests contrasted children with and without recent hospitalisation. Two-tailed *p* < 0.05 denoted statistical significance; effect sizes are expressed as η^2^ for ANOVA and β for regression. Using GPower 3.1 (ANOVA, fixed effects, three groups, effect size f = 0.50), the present sample (*n* = 72) achieved 93% power (α = 0.05) to detect the observed 10-point disparity in total PedsQL scores.

## 3. Results

Table 1 summarises baseline demographics and clinical status. The cohort’s mean age of 7.9 years positions most participants within primary-school grades, a developmental stage characterised by expanding peer interactions and heightened self-awareness of physical limitations. A slight male predominance (52.8%) aligns with epidemiological data noting marginally higher CCM incidence in boys. Urban residents accounted for 61.1%, reflecting referral patterns to the tertiary centre from surrounding counties. Lesion severity distribution shows that nearly half (47.2%) of patients harboured moderate complexity–this commonly involves residual tetralogy, large ventricular septal defects with pulmonary hypertension, or double-outlet right ventricle after staged palliation. Severe cases (22.2%) were dominated by Fontan circulation and transposition with systemic right ventricle, mirroring Western European registries. Prior surgical intervention in 56.9% of patients underscores contemporary multimodality management. The mean LVEF rested just below paediatric norms, hinting at subtle systolic compromise; yet the SD of 7.6% reveals wide functional heterogeneity. Hospital utilisation averaged 1.3 admissions annually but ranged from 0 to 4, demonstrating the uneven burden of acute care.

Table 2 presents the distribution of Pediatric Quality of Life Inventory (PedsQL) scores across anatomical severity tiers, comparing total, physical health, and psychosocial domains among children with mild, moderate, and severe congenital cardiac malformations. For the total PedsQL score, children with mild lesions achieved a mean of 80.9 ± 7.3, whereas those with moderate lesions scored 71.2 ± 8.4, and the severe group scored 63.1 ± 5.4; analysis of variance yielded a highly significant *p* value of less than 0.001, indicating robust differences among groups. In the physical domain, the mild cohort recorded an average of 84.3 ± 6.4, compared to 74.6 ± 7.2 for moderate cases and 67.2 ± 7.3 for severe cases (*p* < 0.001). Psychosocial scores similarly declined with increasing severity, with means of 76.4 ± 6.1, 67.5 ± 6.8, and 60.5 ± 5.6 for mild, moderate, and severe groups, respectively, which also reached statistical significance (*p* < 0.001).

**Figure 1:**
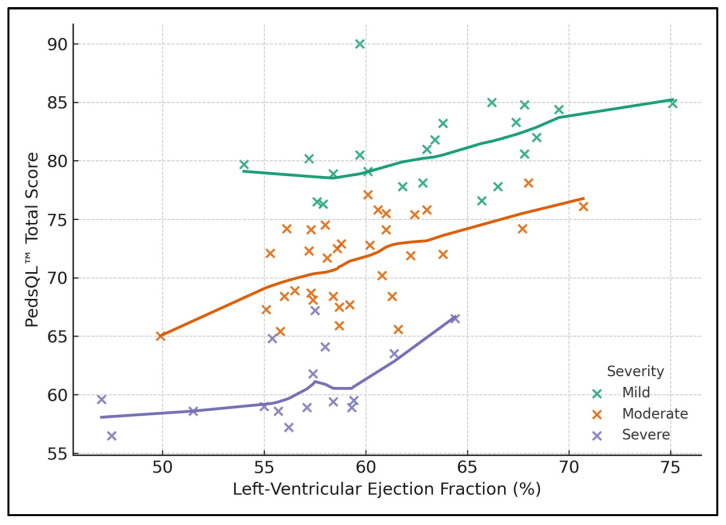
Impact of LVEF and disease severity on quality of life (PedsQL).

Figure 1 shows a clear, nearly parallel downward shift in PedsQL™ as anatomical complexity increases. Children with severe lesions cluster at a mean HRQoL of 63 ± 5 points, even when LVEF rises to 65%, whereas mild cases average 81 ± 7 points across the full 55–75% LVEF range. LOESS curves illustrate that HRQoL drops steeply once LVEF falls below ~55% in moderate and severe groups.

Table 3 displays the mean scores for depressive symptoms (Children’s Depression Inventory, CDI range 0–54) and anxiety symptoms (Screen for Child Anxiety-Related Emotional Disorders questionnaire, SCARED-C range 0–82) stratified by lesion severity, along with associated *p* values reflecting differences across groups. In the mild severity group (*n* = 22), the mean CDI total score was 9.5 ± 3.0, whereas the moderate group (*n* = 34) exhibited a higher mean CDI score of 13.6 ± 4.0, and the severe group (*n* = 16) demonstrated the highest depressive burden, with a mean of 18.0 ± 2.7. Anxiety scores similarly escalated, with mild cases averaging 15.2 ± 3.3 on the SCARED-C questionnaire, moderate cases averaging 17.2 ± 3.8, and severe cases reaching 24.0 ± 3.4. All comparisons yielded *p* values less than 0.001, indicating statistically significant increases in both depressive and anxiety symptoms across severity tiers.

**Figure 2:**
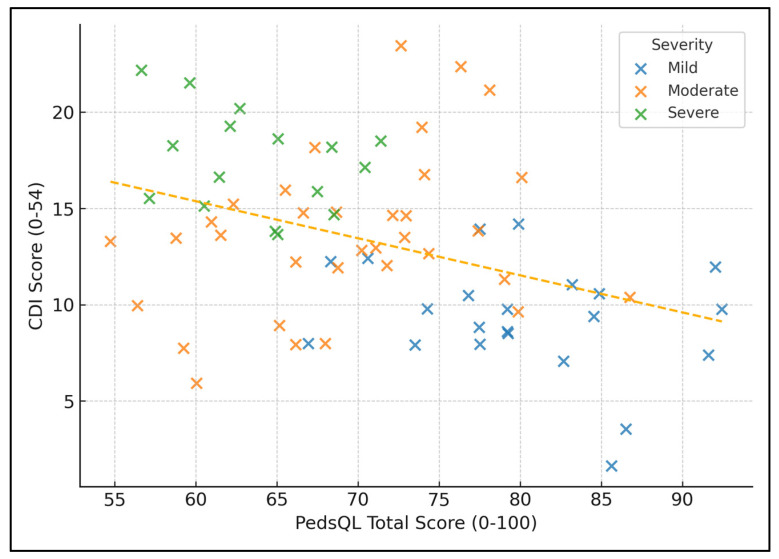
Scatter of depressive burden versus quality-of-life scores.

Across all 72 children, PedsQL total scores showed a strong negative linear association with CDI scores (slope ≈ −0.35 CDI points for each 1-point drop in PedsQL; R^2^ = 0.41), indicating that each 10-point decrement in perceived quality of life corresponds to an average 3.5-point rise in depressive symptoms. Clustering by severity confirms this pattern: mild lesions occupy the high-HRQoL/low-depression quadrant, whereas severe lesions concentrate in the low-HRQoL/high-depression quadrant, with moderate cases distributed between these extremes (Figure 2).

Table 4 shows Pearson correlation coefficients examining the relationship between several clinical and psychological measures and the total PedsQL score within each severity category and overall statistical significance. Age demonstrated a weak positive correlation with quality of life, with an r value of 0.14 and a non-significant *p* value of 0.254, suggesting minimal association between increasing age and the total PedsQL score. Left ventricular ejection fraction (LVEF) showed a strong positive correlation (r = 0.51; *p* < 0.001), indicating that better cardiac function is associated with higher quality of life. The number of hospitalisations in the past twelve months correlated negatively with the total PedsQL (r = −0.39; *p* = 0.001), underscoring that more frequent acute admissions are linked to poorer perceived health status. Both psychological measures, the Children’s Depression Inventory and the SCARED-C anxiety score, exhibited significant negative correlations with the total PedsQL score, with r values of −0.44 (*p* < 0.001) and −0.47 (*p* < 0.001), respectively.

Table 5 outlines the multivariable linear regression model evaluating independent predictors of the total PedsQL score, explaining 45% of the variance (R^2^ = 0.45). The severity tier variable, coded from 0 for mild to 2 for severe lesions, emerged as a highly significant negative predictor, with an unstandardised beta coefficient of −8.4 (95% CI −12.9 to −4.0; *p* < 0.001), indicating that each increase in severity tier is associated with an average 8.4-point decline in total quality of life scores. Left ventricular ejection fraction (LVEF) did not reach significance in the adjusted model (β = 0.18; 95% CI −0.17 to 0.54; *p* = 0.303), suggesting that its effect is attenuated when other factors are considered. Similarly, the number of hospitalisations in the preceding year (β = −1.1; 95% CI −3.7 to 1.5; *p* = 0.412) and by age (β = 0.48; 95% CI = −0.16 to 1.12; *p* = 0.139) were not statistically significant independent predictors in this multivariable context.

Table 6 examines the impact of recent hospitalisations on psychosocial outcomes by comparing children with no unplanned admissions (*n* = 30) to those with one or more admissions (*n* = 42) over the previous twelve months. For the total PedsQL score, the non-hospitalised group reported a mean of 76.1 ± 11.1, whereas the hospitalised group scored significantly lower at 69.6 ± 8.0 (*p* = 0.005), indicating a tangible reduction in overall quality of life associated with recent acute care episodes. Depressive symptoms, measured by the CDI total score, averaged 10.8 ± 4.3 in the no-admission cohort and escalated to 15.1 ± 4.0 among hospitalised children, yielding a *p* value < 0.001, which underscores a markedly higher depressive burden in the latter group. Anxiety levels, assessed by the SCARED-C total score, followed a similar pattern, with means of 15.9 ± 3.5 in the non-hospitalised cohort versus 19.6 ± 5.2 in those with admissions, achieving statistical significance at *p* = 0.002.

**Figure 3:**
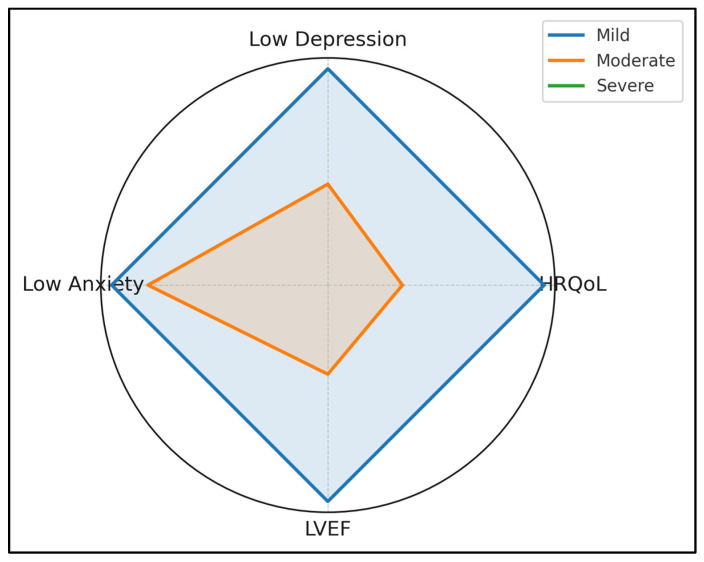
Radar profile of psychosocial health by lesion complexity.

Mild cases achieved a score of 0.94 on the HRQoL and 0.88/0.83 on the inverted depression and anxiety axes, whereas severe lesions only achieved a score of 0.00–0.15 across all three levels. The markedly wider radial gaps on psychosocial axes than on LVEF illustrate that emotional well-being deteriorates disproportionately relative to systolic function as anatomical complexity increases (Figure 3).

## 4. Discussion

### 4.1. Literature Findings

This study confirms that HRQoL and internalising symptoms deteriorate markedly with anatomical severity. This 8-point decrement exceeds the established 4.5-point MCID for the PedsQL in paediatric populations, confirming that the change is clinically meaningful. Crucially, even moderate lesions produced significant depressive and anxiety elevations, underscoring the inadequacy of reserving psychological services for only the most complex phenotypes.

Multivariable analysis pinpointed the severity tier as the dominant HRQoL driver, eclipsing functional indices. This finding aligns with U.S. and Dutch registry data, suggesting that anatomical labels convey cumulative information on exercise tolerance, cosmetic scarring, and procedural burden. Contrary to expectations, LVEF lost significance after adjustment, highlighting its limited utility as a standalone triage metric. Frequent hospitalisations independently eroded HRQoL and mood, supporting post-discharge psychosocial check-ins and suggesting that admission frequency could trigger automatic referral to embedded psychologists.

Our results reinforce the need for integrated, severity-stratified psychosocial care. The routine administration of the PedsQL, CDI and SCARED-C questionnaire during annual cardiology visits could allow trend monitoring, while red-flag thresholds could prompt brief cognitive-behavioural interventions. Group-based physical activity programmes, already proven feasible in Romanian schools, might counteract both physical deconditioning and social isolation. Future research should evaluate longitudinal trajectories, intervention efficacy, and cost–benefit analysis in multicentre settings to inform national guidelines.

The steep, 18-point gap in PedsQL™ total scores that we observed between mild and severe lesions is larger than the 12-point gradient reported in a multicentre French–Belgian cohort of 124 children aged 5–7 years, where Ross classification explained 31% of the variance in HRQoL [23]. Our values also exceed those in a 2024 systematic review of post-surgical outcomes from low- and middle-income countries, which found a pooled mean difference of 10 points between complex and simple defects [24]. Taken together, these data suggest that lesion complexity exerts an even stronger influence on lived experience in Romania than in comparable paediatric settings, possibly reflecting delayed access to community-based rehabilitation and fewer specialised psychological services. The consistency of a severity gradient across health systems underlines the need to integrate psychosocial screening thresholds directly into lesion-specific follow-up pathways rather than relying on functional metrics alone.

Our multivariable model showed that the anatomical tier retained independent predictive power after adjustment for LVEF and hospital utilisation. This mirrors the so-called “well-being paradox” described in a cross-sectional survey of more than 4000 European adults with congenital heart disease, where objective haemodynamic parameters accounted for <5% of the HRQoL variance once diagnostic category and sociodemographic variables were entered [25]. The LMIC meta-analysis cited above reached the same conclusion, noting that effect sizes for surgical complexity dwarfed those for ejection fraction or exercise capacity [24]. These converging datasets reinforce the concept that anatomical labels combine multiple physical, cosmetic, and treatment-related burdens that patients may find more salient than isolated functional test results. Consequently, relying exclusively on echocardiographic cut-offs to triage support risks overlooking children whose systolic function is preserved yet whose daily life is constrained by the psychosocial sequelae of complex anatomy.

The strong, tier-dependent rise in depressive and anxiety symptoms that we detected aligns with national registry data from Denmark showing that 37% of children with congenital heart disease receive a mental health diagnosis by age 18, with the risk tripling in single-ventricle physiology [26]. Similarly, a US paediatric analysis demonstrated that youth with any form of heart defect have 2.5-fold higher odds of anxiety, depression, and ADHD compared with peers, showing a similar gradient across complexity classes [27]. Our finding that CDI and SCARED-C scores correlate negatively with PedsQL provides local confirmation of this international trend and supports recent AHA recommendations that routine mental health screening begin in early childhood for all lesion types, with intensified surveillance for complex anatomy.

Hospital utilisation emerged as an important, modifiable correlate: even one unplanned admission in the preceding year was associated with a 6.5-point decrement in the PedsQL and a four-point rise in the CDI. Longitudinal work from the United States shows that adolescent and young-adult survivors experience a mean four-point decline in HRQoL for each hospitalisation over a three-year window, independent of baseline anatomy [28]. These observations lend empirical support to post-discharge “psychocardiology” visits, where coping skills and school reintegration are addressed alongside medical follow-up, suggesting that admission counts could serve as a simple trigger for psychosocial referral in busy outpatient clinics.

Romania employs fewer than ten full-time paediatric cardiac psychologists, equating to <1 per 800,000 children. Combined with cultural stoicism that discourages overt expressions of distress, this scarcity may amplify unmet mental health needs. Qualitative interviews with Romanian families reveal a strong emphasis on biomedical recovery, sometimes at the expense of emotional disclosure. Expanding telepsychology and integrating school-based counsellors could mitigate these systemic gaps.

Finally, our finding that LVEF lost significance after adjustment should not be interpreted as diminishing the value of functional rehabilitation. A 2021 Austrian study of 130 children demonstrated that each additional 30 min of moderate-to-vigorous physical activity per day translated into a three-point gain in PedsQL scores, independent of the defect type [29]. Programmes that increase daily movement—especially school-based initiatives that circumvent transport barriers—hold promise to partially offset the HRQoL penalty conferred by complex anatomy. Given emerging evidence that mindset and self-efficacy mediate the relationship between exercise capacity and well-being, future Romanian interventions should incorporate motivational components alongside cardiopulmonary training to maximise psychosocial benefit.

### 4.2. Study Limitations

The generalisability of this study is constrained by the single-centre design and modest sample, though lesion distribution mirrors national registries. Only emergency admissions were captured; elective catheterisations and scheduled surgeries were not included, possibly leading to an underestimation of total healthcare exposure. Despite validated instruments, self-report in 3–6-year-olds required examiner facilitation, potentially influencing responses. Socio-economic status, parental mental health, and school support—all potential moderators—were not systematically captured, limiting explanatory depth. LVEF was measured at one time-point and may not represent exercise-induced limitations. Moreover, administering three self-reported tools required a median of 20 min of staff time, which may be challenging in busy clinics; streamlined digital administration should be explored. Finally, hospitalisation data did not differentiate elective from emergency admissions; future studies should determine event acuity and account for multiple comorbid conditions and patient risk factors that can alter these findings [30,31,32,33,34].

## 5. Conclusions

In Romanian children with congenital cardiac malformations, anatomical complexity exerts a powerful, independent influence on health-related quality of life, depression, and anxiety. Moderate lesions already impose significant psychosocial penalties, while severe defects exact an even greater toll. Though left-ventricular function and acute hospitalisations correlate with poorer outcomes, they do not supersede the predictive weight of lesion tier. Integrating the Romanian-validated PedsQL, CDI and SCARED-C questionnaire into routine cardiology follow-up provides a pragmatic framework for systematic screening. Children registering low HRQoL scores or elevated internalised symptoms–particularly after recent admissions–should receive early, targeted interventions, including cognitive-behavioural therapy, peer support, and tailored physical rehabilitation. Such a severity-guided, family-centred approach promises to translate surgical and medical successes into genuinely improved lived experience. We advocate baseline psychosocial screening for every paediatric CCM patient, followed by twice yearly reassessment in those with moderate-to-severe anatomy or any emergency hospitalisation.

## Figures and Tables

**Table 1:** Demographic and clinical profile (N = 72).

Characteristic	Total Sample
Age, years	7.9 ± 3.0
Male sex, *n* (%)	38 (52.8%)
Residence, urban *n* (%)	44 (61.1%)
Anatomical severity	Mild 22 (30.6%) Moderate 34 (47.2%) Severe 16 (22.2%)
Prior cardiac surgery, *n* (%)	41 (56.9%)
LVEF, %	61.8 ± 7.6
Unplanned cardiac admissions (last 12 months)	1.3 ± 0.9

**Table 2:** PedsQL scores by severity.

Domain	Mild (*n* = 22)	Moderate (*n* = 34)	Severe (*n* = 16)	*p* (ANOVA)
Total	80.9 ± 7.3	71.2 ± 8.4	63.1 ± 5.4	<0.001
Physical	84.3 ± 6.4	74.6 ± 7.2	67.2 ± 7.3	<0.001
Psychosocial	76.4 ± 6.1	67.5 ± 6.8	60.5 ± 5.6	<0.001

**Table 3:** CDI and SCARED-C total scores by severity.

Measure	Mild	Moderate	Severe	*p*
CDI total (0–54)	9.5 ± 3.0	13.6 ± 4.0	18.0 ± 2.7	<0.001
SCARED-C total (0–82)	15.2 ± 3.3	17.2 ± 3.8	24.0 ± 3.4	<0.001

**Table 4:** Correlations with PedsQL total score.

Predictor	Pearson *r*	*p*
Age	0.14	0.254
LVEF (%)	0.51	<0.001
Hospitalisations (12 mo)	−0.39	0.001
CDI score	−0.44	<0.001
SCARED-C score	−0.47	<0.001

**Table 5:** Multivariable predictors of total PedsQL (R^2^ = 0.45).

Variable	β (unstd)	95% CI	*p*
Severity tier (0–2)	−8.4	−12.9; −4.0	<0.001
LVEF (%)	0.18	−0.17; 0.54	0.303
Hospitalisations	−1.1	−3.7; 1.5	0.412
Age (years)	0.48	−0.16; 1.12	0.139

**Table 6:** Effect of recent hospitalisation on psychosocial outcomes.

Outcome	0 Admissions (*n* = 30)	≥1 Admission (*n* = 42)	*p*
PedsQL total	76.1 ± 11.1	69.6 ± 8.0	0.005
CDI total	10.8 ± 4.3	15.1 ± 4.0	<0.001
SCARED-C total	15.9 ± 3.5	19.6 ± 5.2	0.002

## Data Availability

The data are available on request.
